# Immunogenicity and reactogenicity of a booster dose of a typhoid conjugate vaccine (TCV) in Malawian pre-school children

**DOI:** 10.1016/j.eclinm.2025.103100

**Published:** 2025-02-12

**Authors:** Nginache Nampota-Nkomba, Osward M. Nyirenda, Shrimati Datta, Victoria Mapemba, Priyanka D. Patel, Theresa Misiri, Felistas Mwakiseghile, John M. Ndaferankhande, Bright Lipenga, Jennifer Oshinsky, Marcela F. Pasetti, Leslie P. Jamka, Melita A. Gordon, Matthew B. Laurens, Kathleen M. Neuzil, TyVAC team

**Affiliations:** a Blantyre Malaria Project, Kamuzu University of Health Sciences, Blantyre, Malawi; b Center for Vaccine Development and Global Health, University of Maryland School of Medicine, Baltimore, MD, United States; c Malawi-Liverpool-Wellcome Program, Kamuzu University of Health Sciences, Blantyre, Malawi; d Fogarty International Center, National Institute of Health, Bethesda, MD, United States

**Keywords:** Typhoid conjugate vaccine, *Salmonella enterica*, *Salmonella* typhi, Typhoid fever, Malawi, Africa, Booster dose, Immunogenicity, Tetanus, Enteric fever, Invasive bacterial infection

## Abstract

**Background:**

We assessed persistence of typhoid immunity conferred by Vi polysaccharide-tetanus toxoid (Vi-TT) conjugate vaccine (TCV) four years post-vaccination and immunogenicity of a booster dose of Vi-TT given at age five.

**Methods:**

In 2018, a phase 3 trial of Vi-TT in Malawi randomised children 1:1 to receive Vi-TT or meningococcal capsular group A conjugate vaccine (control). Subsequently, TCV was licensed and recommended in the region. In 2023, children vaccinated at 9–11 months in the original trial received a second (Booster- TCV) or first (1st-TCV) Vi-TT dose, at age five. Serum collected at days 0, 28, and 160–180 days after vaccination was tested for anti-Vi immunoglobulin (Ig)G and IgA, reported as enzyme-linked immunosorbent assay units (EU)/mL. Seroconversion was ≥4-fold rise in antibody titers from day 0 to day 28 post-vaccination. Safety outcomes included adverse events during follow-up.

**Findings:**

We enrolled 136 children: 72 Booster-TCV and 64 1st-TCV. At baseline, anti-Vi IgG geometric mean titers (GMT) were higher in Booster-TCV (18.8 EU/mL, 95% CI 15.2–23.2) than 1st-TCV (5.7 EU/mL, 4.6–7.2) arms. GMT increased significantly between days 0 and 28 in both arms, with higher levels in Booster-TCV (6867.9 EU/mL, 5794.1–8140.6) than 1st-TCV (2912.0 EU/mL, 2429.2–3490.7) arms, representing a 375.7 and 492.6 geometric mean fold rise, respectively. On day 28, all Booster-TCV children, and all but one 1st-TCV child, seroconverted. Similar trends were seen for IgA. Vi-TT reactogenicity was similar between vaccine arms.

**Interpretation:**

This study demonstrates sustained Vi-TT immunogenicity four years post-vaccination at 9–11 months old, and robust immune response following a booster dose at five years of age, informing policy decisions on TCV use in children.

**Funding:**

10.13039/100000865Bill & Melinda Gates Foundation (INV-030857).

**Research in context**

**Evidence before this study**

Since the 2018 World Health Organization recommendation for programmatic use of typhoid conjugate vaccines (TCVs) in high-burden areas and regions with antimicrobial-resistant *Salmonella* Typhi, several sub-Saharan African and Southeast Asian countries have introduced TCVs into routine immunisation programs. These programs target children at nine months of age or in their second year of life to provide long-term protection against typhoid. However, data on long-term protection from TCV and the need for, and timing of, a booster dose in young children are limited. We conducted a PubMed search for clinical trials assessing duration of protection of TCV and immunogenicity from a two-dose TCV regimen. The search terms were “typhoid conjugate vaccine,” “efficacy,” and “immunogenicity”—from January 1, 1980 to July 29, 2024—without language restrictions. For studies on duration of protection, we filtered for children aged < two years old, a minimum of 12 months follow-up, and a control arm. For two-dose immunogenicity, we included all relevant pediatric clinical trials.

We identified three single dose studies of 25 micrograms of Vi polysaccharide tetanus toxoid conjugate vaccine (Vi-TT) conducted in Malawi, Bangladesh, and Nepal. These studies revealed immunogenicity and efficacy waned faster in children vaccinated before the age of two compared to older children. For two-dose immunogenicity, two studies in Indonesia and the Philippines using diphtheria toxin Vi polysaccharide conjugate vaccine, and two studies in Malawi and Nepal using Vi-TT, showed no significant improvement in immune response after two doses given one to six months apart. However, studies in India—with longer intervals of two to three years between doses—indicated better immune responses after the second dose, though these differences were not statistically significant, and the studies did not include unvaccinated control groups.

**Added value of this study**

This is the first study to evaluate long-term immunogenicity of TCV in Africa and the first to examine a booster dose four years after initial TCV vaccination at 9–11 months of age. We found that Vi-TT immunogenicity decays rapidly between day 28 and two + years post-vaccination, then declines more slowly from two to four years post-vaccination, remaining higher than in children who did not receive TCV at 9–11 months. A booster dose of Vi-TT at five years of age produced a robust immune response, higher than the initial response at 9–11 months and higher than a first vaccination at five years of age. Additionally, Vi-TT vaccination boosted tetanus antibody levels.

**Implications of all the available evidence**

Our findings on the robust booster response of Vi-TT at five years of age—following primary vaccination at 9–11 months of age—will inform decisions on booster-dose schedules for countries introducing TCV into routine immunisation programs. These data, along with information on local typhoid epidemiology, antimicrobial resistance, logistical considerations, and cost-effectiveness, are crucial for policy decision-making.

## Introduction

Typhoid fever, a bacterial infection caused by *Salmonella enterica* serovar Typhi (*S*. Typhi), causes over seven million cases and more than 93,000 deaths globally each year.^1^^,^^2^ The World Health Organization (WHO) recommends a single dose of typhoid conjugate vaccine (TCV) in endemic countries through routine immunisation—either at nine months of age or during the second year of life, to protect against typhoid.^3^ A single dose of TCV is safe, immunogenic,^4^, ^5^, ^6^, ^7^, ^8^, ^9^ and 78–85% effective in preventing typhoid fever for approximately two years in children in diverse settings in sub-Saharan Africa and Southeast Asia.^10^, ^11^, ^12^, ^13^, ^14^, ^15^, ^16^, ^17^ In Malawi, children aged nine months to 12 years in a randomised, controlled trial (RCT) of Typbar TCV® (Bharat Biotech International, Hyderabad, India) Vi polysaccharide conjugated to a nontoxic tetanus toxoid (Vi-TT) were followed for at least four years, and efficacy was well-maintained at 78.3% against blood culture-confirmed typhoid fever.^18^

While overall TCV immunogenicity and efficacy are robust, children in endemic countries vaccinated at a young age continue to be exposed to *S*. Typhi. Therefore, understanding age-specific immunogenicity and efficacy is critical to inform immunisation program planning. Though efficacy was demonstrated in all age groups in the Malawi trial, the point estimate was modestly lower (70.6%) in children vaccinated before two years of age. In addition, anti-Vi IgG responses were lower two years post-vaccination in children receiving Vi-TT before age two years versus older children.^4^ Although differences in efficacy and immunogenicity were not statistically significant, the trend was evident, and similar trends of lower immunogenicity in children under two years of age have been observed in other populations.^17^^,^^19^

In this study, we aimed to assess the persistence of typhoid immunity and immunogenicity of a Vi-TT booster dose given at approximately five years of age in a subset of children originally vaccinated with Vi-TT or meningococcal A polysaccharide tetanus toxoid conjugate vaccine (Men-A), at 9–11 months of age as part of the Malawi RCT. Our rationale for administering a booster dose more than four years after the primary dose given at 9–11 months includes the following considerations: programmatically, 9–11 months is when TCV is given in Malawi and several other countries; vaccine responses are age-dependent with lowest responses in the youngest children; demonstrated vaccine protection for four years in this population; feasibility of administering a school entry vaccine dose; and biological plausibility for a robust booster response after a prolonged interval. Given the paucity of data on a TCV booster dose beyond three years after the initial dose, these data will inform decisions regarding the need for—and timing of—booster-dose schedules in countries introducing TCV into routine childhood immunisation programs.

## Methods

### Study design and participants

This booster study was an extension of a larger phase 3, double-blind, RCT. The booster study aimed to determine safety and immunogenicity of a Vi-TT booster dose given at five years of age after primary vaccination at 9–11 months.

The RCT methods have been previously described.^20^ In brief, 28,130 children aged nine months to 12 years were recruited from Ndirande and Zingwangwa townships in Blantyre, Malawi in 2018 and randomised 1:1 to receive either Vi-TT or control Men-A. Follow-up continued until 2022.^12^^,^^18^ A subset of 602 children participated in a safety and immunogenicity sub-study, with serum collected at day 0 (pre-vaccination), day 28, and day 730+ (day 730–1095) post-vaccination.^4^

The booster study took place between January and October 2023, more than four years after primary vaccination. Vaccine used in this study Typbar-TCV is recommended for use by WHO and licensed for use in Malawi. Children were eligible if they were initially vaccinated at 9–11 months of age in the parent RCT. Eligible children were contacted through phone calls, home visits, and community messages. Written informed consent was obtained at the study clinic in Blantyre. Study staff remained blinded to the RCT vaccine assignments.

### Ethics

Ethical approval was secured from the Malawi National Health Science Research Committee (FWA00005976), the University of Liverpool Ethical Review Board (FWA00005266), and the University of Maryland Institutional Review Board (FWA00007145).

### Procedures

All eligible participants received a full dose of Vi-TT consisting of 25 μg of Vi polysaccharide in 0.5 mls. Vi-TT was administered as a single intramuscular injection in the left deltoid on day 0. Participants originally assigned Men-A in the parent RCT received their first dose of Vi-TT (1st-TCV), while those originally assigned Vi-TT received a second dose (Booster-TCV).

After vaccination, participants were observed for 30 min at the study clinic by trained clinicians to monitor for immediate adverse events (AEs). Additional safety assessments were conducted during in-person follow-up visits on days 7, 28, and 160–180 post-vaccination. Serum was collected prior to vaccination (day 0) and 28- and 160–180 days post-vaccination. All samples were tested for anti-Vi immunoglobulin (Ig)G, and anti-Vi IgA using VaccZyme Human anti-*S.* Typhi Vi ELISA kits (The Binding Site Group Ltd, Birmingham, UK). Anti-Vi IgG was measured according to manufacturer's instructions, and anti-Vi IgA following a protocol adapted from the commercial VaccZyme assay.^21^ An optimised in-house ELISA was used to measure anti-tetanus toxoid IgG; the assay was based on a published procedure with brief modifications: microtiter plates were coated with 5 μg/mL of tetanus toxoid overnight and blocked with 5% non-fat dried milk in PBS containing 0.05% Tween 20 (also used as diluent buffer).^22^ WHO Tetanus Antitoxin Standard TE-3 was used as calibrator; titers were calculated by interpolation of OD values in a 4 PL regression curve of TE-3. Testing was conducted at the University of Maryland School of Medicine, Baltimore, Maryland, US.

The Malawi Ministry of Health was planning a national TCV campaign to begin during the follow-up period, administering Vi polysaccharide conjugated to a non-toxic mutant of diphtheria toxin—Vi-CRM 197 (another WHO prequalified TCV), to all children aged nine months to less than 15 years in health facilities, established community outreach sites and mobile sites such as schools, churches, and orphan centers. When participants presented for their day 28 visit, parents were asked not to allow their children to receive an additional TCV dose as part of the campaign. At each visit after the start of the campaign, study staff verified whether study participants had received an additional dose of TCV during the campaign by verbally asking their parents and reviewing their vaccination records. The campaign vaccination was recorded on a special vaccination card and/or in the child's health record book.

### Outcomes

Local and systemic AEs occurring between day 0 and day 7 were solicited and recorded by study clinicians during the day 7 visit, using the same questions as the RCT safety and immunogenicity sub-study to assess safety.^4^ Non-serious unsolicited AEs were collected until day 28 post-vaccination, while severe adverse events (SAEs) were collected for the study duration.

Immunogenicity was assessed by measuring anti-Vi IgG, anti-Vi IgA, and anti-tetanus toxoid serum IgG geometric mean titers (GMT). The day 0 sample was collected to evaluate duration of immunity after original 9-11-month vaccination; day 28 and 160–180 samples were obtained to evaluate the primary or booster response. Seroconversion was defined as a ≥ four-fold rise in anti-Vi titers from day 0 to day 28 and from day 0 to day 160–180 post-vaccination.^4^, ^5^, ^6^, ^7^, ^8^ For tetanus, short-term seroprotection was defined as anti-tetanus IgG of ≥0.1 international units per milliliter (IU/mL) and long-term seroprotection as anti-tetanus IgG of ≥1.0 IU/mL.^6^

### Statistical analysis

Safety outcomes were analysed based on an intention-to-treat (ITT) population, which included all children with post-vaccination safety data according to their randomised group. We compared the proportion of participants in the Booster-TCV and 1st-TCV arms who experienced solicited local and systemic AEs within seven days after vaccination, as well as unsolicited AEs within 28 days after vaccination, and SAEs throughout the study follow-up period.

Immunogenicity outcomes were analysed in the per-protocol population, which included participants whose samples were collected within the allowable window (±five days) for their scheduled day 28 visit and day 160–180 post-vaccination, and the ITT population. We compared log_10_-transformed anti-Vi IgG, anti-Vi IgA, and anti-tetanus IgG titers at each time point within each vaccine arm using a paired t-test and between the vaccine arms using a two-sample t-test with unequal variances. The distribution of each antibody measure, at each specimen collection time point in each vaccine arm, was examined graphically and reported in terms of sample size, geometric mean and corresponding 95% confidence interval (CI), and p-value. For geometric mean calculation, one-half the limit of detection (3.7 EU/mL for anti-Vi IgG, 1.6 EU/mL for anti-Vi IgA, and 3.1 IU/mL for anti-tetanus IgG) was used for titer values below the limit of detection, or zero. SAS Proc Surveymeans was used to compute geometric means and their corresponding 95% confidence intervals. In SAS, the geometric mean is typically calculated by applying a natural logarithm transformation to the data, computing the arithmetic mean of the transformed values, and then exponentiating the result to return to the original scale. To compute the geometric mean fold rise (GMFR), the fold-rise in antibody titer of interest between two time points was calculated for each subject. The GMFR was then calculated as the geometric mean of these fold-rise values. The proportions of participants in each vaccination arm that seroconverted were compared using confidence intervals calculated using Clopper-Pearson exact method.

For participants who were part of the RCT immunogenicity sub-study, a multi-time point immunogenicity analysis was conducted comparing their prior anti-Vi GMT results from day 0 to day 730+ post-primary vaccination at 9–11 months, to day 0 to day 160–180 post booster vaccination at five years of age. This multi-time point immunogenicity subset analysis was performed in the ITT population.

All study analyses were conducted using SAS software, Version 9.4 (Copyright ® 2016 SAS Institute Inc).

### Role of the funding source

The study's funder was not involved in the design, data collection, analysis, interpretation, or manuscript writing of this study.

## Results

**Fig. 1:**
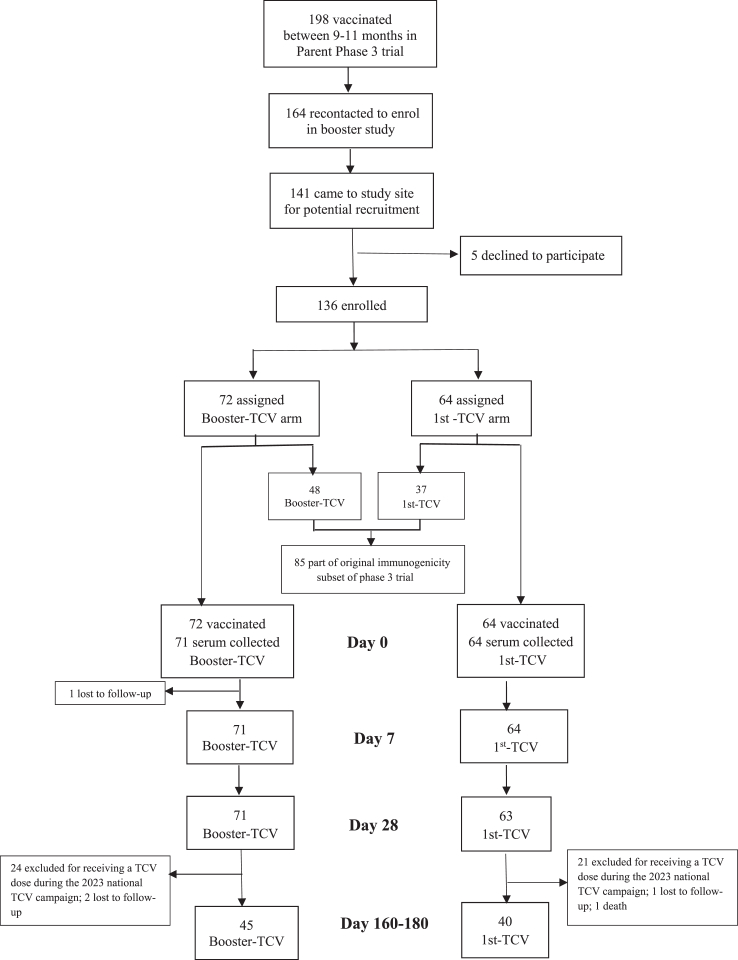
Flow chart.

We recontacted 164 out of 198 (82.8%) children vaccinated between 9 and 11 months in the RCT, and 141 were screened for enrolment. Five declined to participate, resulting in 136 children vaccinated between 26 January and 12 April 2023: 72 in the Booster-TCV arm and 64 in the 1st-TCV arm. Among these, 85 (62.5%) were part of the immunogenicity sub-study of the RCT, with 48 in the Booster-TCV arm and 37 in the 1st-TCV arm (Fig. 1). Both vaccination arms had similar age and gender distributions at enrolment, with a median age of 5.5 years in both and 44.4% girls in the Booster-TCV and 46.9% in the 1st-TCV arm. All but one participant returned for the day 28 visit and had serum collected.

In May 2023, after all day 28 visits were completed, but before day 160–180 visits, the Malawian government conducted a nationwide TCV introduction campaign. A total of 45 participants (24 Booster-TCV and 21 1st-TCV) were vaccinated during the TCV campaign and were therefore excluded from the primary day 160–180 immunogenicity analysis post-hoc (Fig. 1).

Local reactions within 7 days post-vaccination were mostly mild/moderate and occurred at a similar rate in 52/72 Booster-TCV (72.0%, 95% CI 61.0–81.2) and 41/64 1st-TCV (64.1% 95% CI 51.8–74.7) recipients. The most common local reaction was mild/moderate pain. The rate of systemic AEs post-vaccination did not differ significantly between the Booster-TCV (22/72, 30.6%, 95% CI 21.1–42.0) and 1st-TCV (12/64, 18.8%, 95% CI 11.1–30.0) arms. Fever, reported as present or absent, was the most common systemic AE in 26.4% (95% CI 17.6–37.6) Booster-TCV and 10.9% (95% CI 5.4–20.9) 1st-TCV participants (Table 1). One participant from the 1st-TCV arm had severe pain, myalgia, and arthralgia on day 1 post-vaccination, which resolved by day 4. On day 7, one participant in each arm reported fever, but all other AEs had resolved by day 5 post-vaccination.

**Table 1:** Summary of reactogenicity and safety parameters (adverse events) by vaccine arm in the intention to treat population after Vi-TT vaccination given 4 years after receipt of first Vi-TT or Men-A.

	Booster-TCV	1st-TCV
	n = 72	n = 64
Local Reactions at injection site (Day 0–7)		
Pain/tenderness	51 (70.8, 59.5–80.1) Mild 36, moderate 15	40 (62.5, 50.3–73.3) Mild 31, moderate 8, severe 1
Swelling	10 (13.9, 7.7–23.7) Mild 9, moderate 1	10 (15.6, 8.7–26.4) All mild
Erythema	2 (2.8, 0.8–9.6) All mild	3 (4.7, 1.6–12.9) All mild
Any local reaction	52 (72, 61.0–81.2)	41 (64.1, 51.8–74.7)
Systemic Reactions (Day 0–7)		
Fever	19 (26.4, 17.6–37.6)	7 (10.9, 5.4–20.9)
Myalgia	2 (2.8, 0.8–9.6) All mild	1 (1.6, 1.3–8.3) All severe
Arthralgia	1 (1.4, 0.3–7.5) All mild	1 (1.6, 0.3–8.3) All severe
Malaise	7 (9.7, 4.8–18.7) Mild 6, moderate 1	6 (9.4, 4.4–19.0) Mild 5, moderate 1
Any systemic reaction	22 (30.6, 21.1–42.0)	12 (18.8, 11.1–30.0)
Unsolicited adverse events (Day 0–28)		
Related	1 (1.4, 0.04–7.5)	1 (1.6, 0.04–8.4)
Not related	35 (48.6, 36.7–60.7)	36 (56.3, 43.3–68.6)
Any unsolicited adverse event	36 (50.0, 38.0–62.0)	37 (57.8, 44.8–70.1)

Data are n (%, 95% CI). n = number of participants. CI = confidence interval. Vi-TT: Vi polysaccharide tetanus toxoid conjugate vaccine. Men-A: meningococcal polysaccharide tetanus toxoid conjugate vaccine.

No participants experienced an unsolicited AE within the first 30 min after vaccination. Unsolicited AEs after the initial 30 min post-vaccination occurred at a similar rate in Booster-TCV (50.0%, 95% CI 38.0–62.0) and 1st-TCV (57.8%, 95% CI 44.8–70.1) arms. One participant in each arm had an unsolicited AE that occurred the day of vaccination that may have been related to vaccination. The AEs were itchy eyes (Booster-TCV) and diarrhea (1st-TCV), and both resolved without sequalae. There were two unrelated SAEs, one in each vaccination arm. One was a hospitalisation for malaria five months post-vaccination in a Booster-TCV arm participant who did not receive a third dose of TCV as part of the nationwide TCV campaign, that resolved without sequalae, and the other was an acute central nervous system event 18 days post-vaccination in the 1st-TCV arm that resulted in death. The definitive cause of death was not determined but was suspected to be diabetic ketoacidosis or a central nervous system infection and was deemed unrelated to TCV vaccination by an independent pediatrician.

At baseline, the Booster-TCV arm (18.8 EU/mL, 95% CI 15.2–23.2) had higher anti-Vi IgG geometric mean titers (GMT) than the 1st-TCV arm (5.7 EU/mL, 95% CI 4.6–7.2). GMT rose significantly from day 0 to day 28 in both arms, with the Booster-TCV arm reaching 6867.9 EU/mL (95% CI 5794.1–8140.6), which was significantly higher than the 1st-TCV arm at 2912.0 EU/mL (95% CI 2429.2–3490.7). On day 28, all children in the Booster-TCV arm and all but one child in the 1st-TCV arm seroconverted for IgG. The child who did not seroconvert had high anti-Vi titers (426 EU/ml) at baseline and was not included in the day 160–180 visit analysis. By day 160–180, GMT dropped but remained significantly higher than baseline in both arms (Table 2 and Supplemental Figure S1). Two children in the Booster-TCV arm did not seroconvert at day 160–180. Both had detectable anti-Vi titers at baseline (46 EU/mL and 144 EU/mL) and had seroconverted by day 28. However, they did not meet the four-fold rise requirement by day 160–180, with titers of 183 EU/mL and 501 EU/mL, corresponding to 3.98-fold and 3.47-fold increases, respectively. Comparable IgG results were seen in the ITT analysis (Supplemental Table S1).

**Table 2:** Anti-Vi IgG before, 28 and 160–180 days after Vi-TT vaccination given 4 years after receipt of first Vi-TT or Men-A in the per-protocol population.

	Booster-TCV		1st-TCV	
	*n or n/N*		*n or n/N*	
Geometric Mean Titer (GMT, EU/mL)		*GMT (95% CI)*		*GMT (95% CI)*
Day 0	71	18.8 (15.2–23.2)	64	5.7 (4.6–7.2)
Day 28	70	6867.9 (5794.1–8140.6)	60	2912.0 (2429.2–3490.7)
Day 160–180^a^	44	495.2 (381.1–643.3)	39	306.9 (218.7–430.6)
Median (Interquartile range)		*Median (q1, q3)*		*Median (q1, q3)*
Day 0	71	18.1 (12.7, 30.8)	64	3.7 (3.7, 7.5)
Day 28	70	6834.3 (4394.9, 10,822.1)	60	2837.0 (1554.9,4758.2)
Day 160–180^a^	44	439.7 (289.9, 926.4)	39	257.6 (155.9, 453.4)
Geometric Mean Fold Rise (GMFR)				
Day 0–28	69	375.7 (293.2–481.4)	60	492.6 (364.3–666.1)
Day 0 to 160–180^a^	43	25.3 (19.1–33.5)	39	54.4 (37.6–78.7)
Seroconversion ≥ 4-fold increase from		*% (95% CI)*		*% (95% CI)*
Day 0–28	69/69	100.0 (94.8–100.0)	59/60	98.3 (91.1–100.0)
Day 0 to 160–180^a^	41/43	95.4 (84.2–99.4)	39/39	100.0 (91.0–100.0)

n = number of participants. CI = confidence interval. Vi-TT: Vi polysaccharide tetanus toxoid conjugate vaccine. Men-A: meningococcal polysaccharide tetanus toxoid conjugate vaccine. EU: ELISA units.

a Participants who received TCV as part of the Malawi national campaign were excluded.

For IgA, baseline GMT was higher in the Booster-TCV arm than the 1st-TCV arm. Both arms showed significant GMT rises at day 28, with higher values in the Booster-TCV arm (117.7 EU/mL, 95% CI 93.0–148.9) compared to the 1st-TCV arm (95.0 EU/mL, 95% CI 78.5–115.0). All but one participant in the Booster-TCV arm, and all but two in the 1st-TCV arm, seroconverted for IgA at day 28. One participant had non-detectable titers at baseline. The other two had detectable titers (15 EU/ml and 56 EU/ml) and were not included in the day 160–180 analysis. By day 160, GMTs dropped to similar levels in the Booster-TCV and 1st-TCV arms. All remaining Booster-TCV participants seroconverted at the final visit, while one in the 1st-TCV arm did not (Table 3 and Supplemental Figure S2). ITT results demonstrate a similar trend (Supplemental Table S2).

**Table 3:** Anti-Vi IgA before, 28 and 160–180 days after Vi-TT vaccination given 4 years after receipt of first Vi-TT or Men-A in the per-protocol population.

	Booster-TCV		1st-TCV	
	*n or n/N*		*n or n/N*	
Geometric Mean Titer (GMT, EU/mL)		*GMT (95% CI)*		*GMT (95% CI)*
Day 0	71	2.6 (2.2–3.1)	64	1.7 (1.5–1.9)
Day 28	70	127.0 (103.8–155.5)	60	86.8 (68.5–110.0)
Day 160–180^a^	44	60.4 (44.1–82.9)	39	46.5 (34.0–63.8)
Median (Interquartile range)		*Median (q1, q3)*		*Median (q1, q3)*
Day 0	71	1.6 (1.6, 4.3)	64	1.6 (1.6, 1.6)
Day 28	70	125.3 (80.7, 199.5)	60	104.5 (56.5, 151.0)
Day 160–180^a^	44	57.0 (34.5, 108.7)	39	49.6 (24.3, 73.3)
Geometric Mean Fold Rise (GMFR)		*GMFR (95% CI)*		*GMFR (95% CI)*
Day 0–28	69	49.8 (39.3–62.9)	60	51.7 (38.7–69.1)
Day 0 to 160–180^a^	43	25.5 (19.3–33.8)	39	29.2 (21.2–40.2)
Seroconversion ≥ 4-fold increase from		*% (95% CI)*		*% (95% CI)*
Day 0–28	68/69	98.6 (92.2–100.0)	58/60	96.7 (88.5–99.6)
Day 0 to 160–180^a^	43/43	100.0 (91.8–100.0)	38/39	97.4 (86.5–100.0)

n = number of participants. CI = confidence interval. Vi-TT: Vi polysaccharide tetanus toxoid conjugate vaccine. Men-A: meningococcal polysaccharide tetanus toxoid conjugate vaccine. EU: ELISA units.

a Participants who received TCV as part of the Malawi national campaign were excluded.

At day 0, most children (76.1% Booster-TCV and 84.4% 1st-TCV) had short-term tetanus seroprotection (≥0.1 IU/mL), which increased to 100% after Vi-TT vaccination. Fewer children had long-term seroprotection (≥1.0 IU/mL) at baseline, but proportions increased after vaccination to 94.3% and 95% in the Booster-TCV and 1st-TCV arms at day 28, and 68.2% and 74.4% at day 160–180, respectively (Table 4 and Supplemental Figure S3).

**Table 4:** Anti-Tetanus IgG before, 28 and 160–180 days after Vi-TT vaccination given 4 years after receipt of first Vi-TT or Men-A in the per-protocol population.

	Booster-TCV		1st-TCV	
	*n or n/N*		*n or n/N*	
Geometric Mean Titer (GMT,IU/mL)		*GMT (95% CI)*		*GMT (95% CI)*
Day 0	71	0.2 (0.2–0.3)	64	0.3 (0.2–0.4)
Day 28	70	5.3 (4.1–6.8)	60	7.3 (5.4–9.8)
Day 160–180^a^	44	1.4 (1.0–1.9)	39	2.2 (1.5–3.1)
Median (Interquartile range)		*Median (q1, q3)*		*Median (q1, q3)*
Day 0	71	0.2 (0.1, 0.4)	64	0.3 (0.1,0.6)
Day 28	70	5.6 (3.5, 9.8)	60	8.0 (4.0, 14.3)
Day 160–180^a^	44	1.3 (0.8, 2.3)	39	2.8 (1.0, 5.3)
Short term immunity ≥0.1 IU/mL		*GMT (95% CI)*		*GMT (95% CI)*
Day 0	54/71	76.1 (64.5–85.4)	54/64	84.4 (73.1–92.2)
Day 28	70/70	100.0 (94.9–100.0)	60/60	100.0 (94.0–100.0)
Day 160–180^a^	44/44	100.0 (92.0–100.0)	39/39	100.0 (91.0–100.0)
Long term immunity ≥1 IU/mL		*GMT (95% CI)*		*GMT (95% CI)*
Day 0	6/71	8.5 (3.2–17.5)	8/64	12.5 (5.6–23.2)
Day 28	66/70	94.3 (86.0–98.4)	57/60	95.0 (86.1–99.0)
Day 160–180^a^	30/44	68.2 (52.4–81.4)	29/39	74.4 (60.7–88.1)

n = number of participants. CI = confidence interval. Vi-TT: Vi polysaccharide tetanus toxoid conjugate vaccine. Men-A: meningococcal polysaccharide tetanus toxoid conjugate vaccine. IU: International units.

a Participants who received TCV as part of the Malawi national campaign were excluded.

**Fig. 2:**
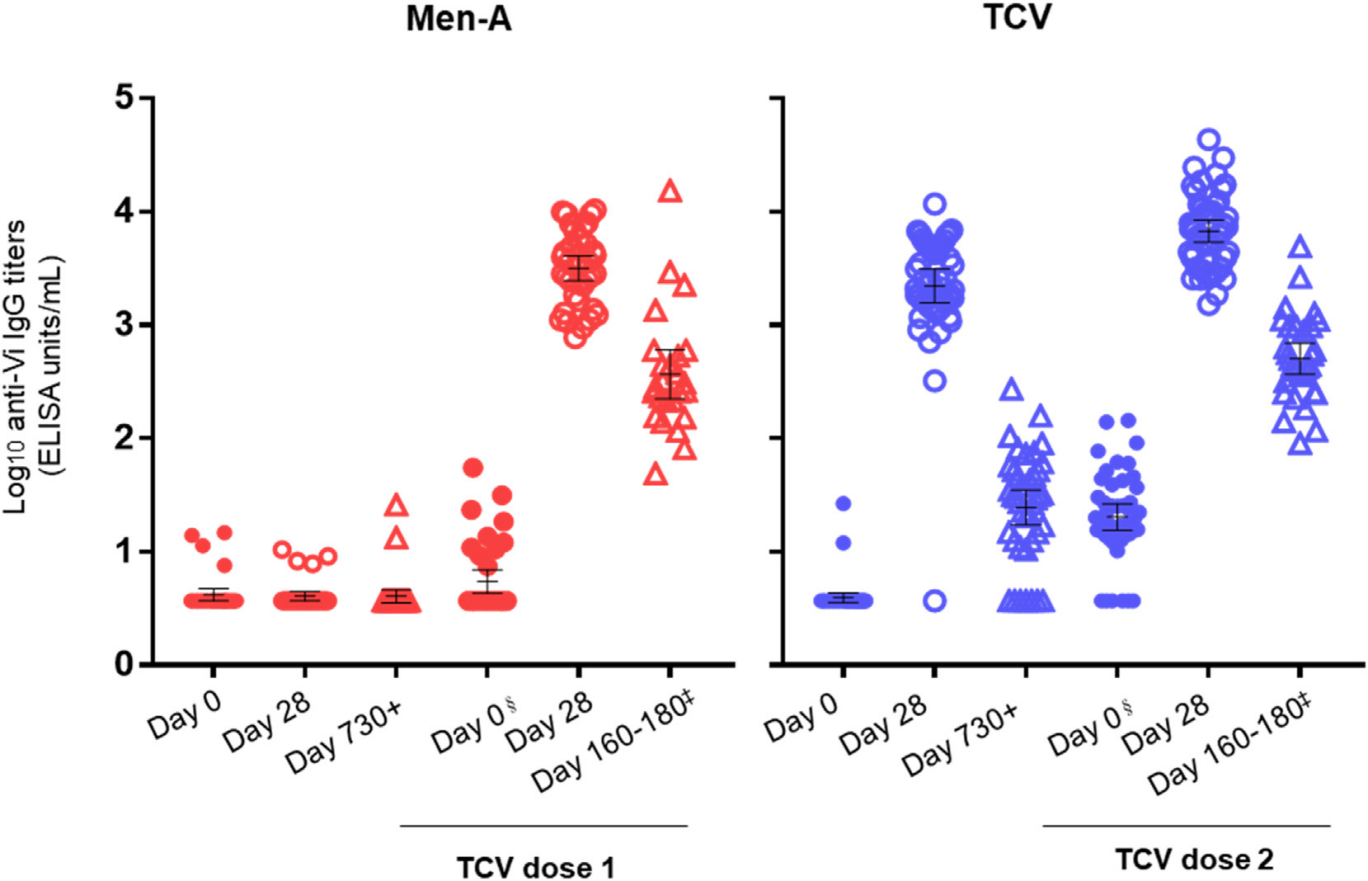
Anti-Vi IgG before and after first Men-A or Vi-TT vaccination and before and after Vi-TT vaccination given 4 years after receipt of first Vi-TT or Men-A in the intention to treat population. ^§^TCV dose 1 and dose 2 received 2+ years post Day 730+ timepoint. ^‡^Participants who received a TCV dose during the 2023 national campaign have been excluded. Vi-TT: Vi polysaccharide tetanus toxoid conjugate vaccine. Men-A: meningococcal polysaccharide tetanus toxoid conjugate vaccine.

Table 5 and Fig. 2 present a multi-time point anti-Vi IgG analysis of children who were part of the RCT immunogenicity sub-study. At day 0, before the initial phase 3 vaccination at 9–11 months of age, Booster-TCV and 1st-TCV participants had similarly low anti-Vi GMT. After Vi-TT vaccination, as previously reported, Booster-TCV participants' GMT rose to 2221.2 EU/ml (95% CI 1573.0–3136.7) at day 28 post-initial vaccination, then dropped to 24.6 EU/ml (95% CI 17.3–35.0) at day 730+ post-initial vaccination, still significantly higher than baseline. At enrolment into the booster study, Booster-TCV arm GMT were 20.2 EU/ml (95% CI 15.5–26.4). Following booster vaccination, GMT increased to 6726.7 EU/ml (95% CI 5370.5–8425.5) at day 28 post-booster vaccination. Conversely, participants in the 1st-TCV arm maintained low anti-Vi IgG GMT from 9 to 11 months until their first TCV vaccination at age 5 years, where GMT rose to 3168.2 EU/ml (95% CI 2460.2–4079.9) at day 28. All 85 children seroconverted by day 28 of the booster study, maintaining seroconversion at day 160–180 post-vaccination.

**Table 5:** Anti-Vi IgG before and after first Men-A or Vi-TT vaccination and before and after Vi-TT vaccination given 4 years after receipt of first Vi-TT or Men-A in the intention to treat population.

	Booster-TCV		1st-TCV	
	*N or n/N*		*n*	
Geometric Mean Titer (GMT, EU/mL)		*GMT (95% CI)*		*GMT (95% CI)*
Initial Vaccination		TCV		Men-A
Day 0	48	4.0 (3.6–4.3)	37	4.2 (3.7–4.7)
Day 28	48	2221.2 (1573.0–3136.7)	37	4.1 (3.7–4.5)
Day 730+	41	24.6 (17.3–35.0)	35	4.1 (3.6–4.6)
Second Vaccination		TCV		TCV
Day 0^b^	47	20.2 (15.5–26.4)	37	5.5 (4.3–7.0)
Day 28	47	6726.7 (5370.5–8425.5)	36	3168.2 (2460.2–4079.9)
Day 160–180^a^	32	504.8 (369.4–689.9)	25	369.2 (223.9–608.9)
Median (Interquartile range)		*Median (q1, q3)*		*Median (q1, q3)*
Initial Vaccination		TCV		Men-A
Day 0	48	3.7 (3.7, 3.7)	37	3.7 (3.7, 3.7)
Day 28	48	2326.5 (1522.0, 4900.6)	37	3.7 (3.7, 3.7)
Day 730+	41	30.2 (12.7, 52.0)	35	3.7 (3.7, 3.7)
Second Vaccination		TCV		TCV
Day 0^b^	47	18.1 (14.1, 36.8)	37	3.7 (3.7, 7.5)
Day 28	47	6656.6 (3935.1, 11,482.0)	36	3395.9 (1738.1, 5517.5)
Day 160–180^a^	32	439.7 (286.2, 969.2)	25	283.7 (212.6, 533.2)
Geometric Mean Fold Rise (GMFR)		*GMFR (95% CI)*		*GMFR (95% CI)*
Day 0 (initial) to 28 (second vaccination)	47	1700.3 (1348.9–2143.3)	36	754.7 (574.7–991.1)
Day 0 (initial) to 160-180 (second vaccination)^a^	32	128.3 (90.1–182.7)	25	91.9 (54.9–153.9)
Seroconversion ≥ 4-fold increase from		*% (95% CI)*		*% (95% CI)*
Day 0 (initial) to 28 (second vaccination)	47/47	100.0 (92.5–100.0)	36/36	100.0 (90.3–100.0)
Day 0 (initial) to 160-180 (second vaccination)^a^	32/32	100.0 (89.1–100.0)	25/25	100.0 (86.3–100.0)

n = number of participants. CI = confidence interval. Vi-TT: Vi polysaccharide tetanus toxoid conjugate vaccine. Men-A: meningococcal polysaccharide tetanus toxoid conjugate vaccine. EU: ELISA units.

a Participants who received TCV as part of the Malawi national campaign were excluded.

b Second vaccination received 2+ years post Day 730+ timepoint.

## Discussion

Among Malawian children who received a primary Vi-TT vaccine at 9–11 months of age, Vi-specific antibody levels declined significantly by four years post-vaccination but remained higher than the antibody levels in children who never received Vi-TT. A second dose of Vi-TT at the four-year time point resulted in a robust antibody response that was higher than the response in children receiving Vi-TT for the first time. The response to the booster dose of TCV was consistent with a memory response since it exceeded that reported after the initial vaccination in these children.^4^

Several studies have assessed the immunogenicity of a two-dose primary schedule of TCV, or a booster dose given at intervals shorter than the four-year interval in our study. For instance, a Vi polysaccharide diphtheria toxoid conjugate vaccine (Vi-DT) trial in Indonesia showed no difference in immunogenicity between one and two doses given four weeks apart to children aged two to five years.^23^ A study in Malawi found that a two-dose schedule of Vi-TT at 9 and 15 months did not outperform a single dose schedule.^9^ In Nepal, a two-dose schedule at 9 and 15 months elicited a stronger response compared to a 12- and 15-month schedule, though not significantly different from a single dose.^24^ A Vi-DT trial in the Philippines indicated a booster given two years after the primary vaccination produced a more robust response than one given after six months, but no significant difference was observed between primary and booster vaccination during both time-intervals.^25^ Additionally, in India, a booster dose of Vi-TT administered two years after a single dose yielded a more robust response than a single dose alone for up to seven years post-vaccination.^26^^,^^27^ However, this study was not randomised, and children in all arms had a high degree of background exposure to *S*. Typhi. For our cohort, as part of the main clinical trial, all children were under passive surveillance for typhoid fever. During the four-year follow-up period, no cases of laboratory-confirmed typhoid fever were identified among the children enrolled in this booster study.^18^ Therefore the robust immunologic response seen with the second dose in our study is likely due to the four-year interval and represents a long-lasting immune memory and a humoral booster effect.^22^

Despite the drop in immunogenicity in our Malawian population, efficacy in this age group was high at 74.4% at two years and 70.6% at four years in the RCT.^12^^,^^18^ These findings are encouraging and suggest that even low levels of antibodies may be protective. In a cluster-randomised trial of Vi-TT in Bangladesh, children initially vaccinated between 9 and 11 months of age had a reduction in immunogenicity to 10 EU/ml at five years post-vaccination.^17^ However, in Bangladeshi children, the drop in immunogenicity was accompanied by a drop in vaccine effectiveness over time. The difference in the results in Bangladesh and Malawi may be attributed to differences in local typhoid fever burden. Typhoid fever incidence in Bangladesh is 1135 per 100,000 person-years, compared to 444 per 100,000 in Malawi.^28^ Therefore, Bangladeshi children may have more frequent and/or higher inoculum exposures to *S*. Typhi and may need higher antibody titers for protection than Malawian children.

It was concerning that some children in this study did not have immunologic protection against tetanus at five years of age. Presumably, most children had received a tetanus-containing vaccine during routine vaccination visits at 6, 10, and 14 weeks of age. As part of this booster study, at 9–11 months of age, all received a vaccine conjugated to tetanus toxoid—either Vi-TT or Men-A. Still, approximately 30% of children were not seroprotected at age 5. At the booster study visit, Vi-TT boosted the tetanus antibody response, ensuring all children had short-term protection (≥0.1 IU/mL) for at least five months post-vaccination, and increased the proportion with long-term protection (≥1.0 IU/mL) to over 68%. The WHO recommends three primary doses of tetanus-containing vaccine in the first six months of life, followed by three booster doses at 12–23 months, four to seven years, and nine to 15 years to ensure protection through adolescence and adulthood.^29^ While children in higher-income countries typically follow this vaccination schedule, children in lower-income countries, like Malawi, often receive only three doses, completing their schedule by 14 weeks of age.^30^ Therefore, Vi-TT administration at school age has the added benefit of boosting tetanus immunity in children who reside in regions where routine booster vaccinations are not available.

The decision on whether—and when—to administer a booster dose is complex. For example, the need for a booster dose will be more compelling in regions where typhoid incidence is higher than Malawi, where antimicrobial resistance is extensive, and/or where typhoid fever peaks in later childhood. Cost-effectiveness analyses will be important, as the number of cases prevented per dose is likely lower for a booster than for a primary vaccination. Additionally, the choice between homologous and heterologous boosting will be influenced by supply and cost considerations. Currently, three types of TCVs are available: Vi-TT, Vi-DT, and Vi-CRM, and Vi-CRM was used during Malawi's vaccination campaigns and is now part of the country's Expanded Program on Immunization. We only tested homologous boosting in our study. Testing of a Vi-CRM booster following a Vi-TT primary dose is on-going in Burkina Faso, with results expected in 2025. Heterologous priming and boosting has been an effective strategy for other vaccines.^31^

In Malawi, while children are protected against typhoid for the first four years post-vaccination, the duration of this protection beyond four years remains uncertain, and it is critical that post-vaccination surveillance continues.^32^ Given that there is no clear correlate of protection for typhoid fever, inferring whether a higher GMT is needed for increased protection will be challenging. Approaches could include comparing GMT levels in children who contract typhoid, compared to those who do not, or evaluating breakthrough infections in populations stratified by GMT. Ongoing studies examining anti-Vi immunogenicity in Vi-TT vaccinated children with and without blood culture-confirmed typhoid fever aim to investigate potential correlates of protection further. While more evidence is needed, it is clinically plausible that a booster Vi-TT dose would extend protection against typhoid fever and administration is safe. Additional public health benefits of a booster dose include boosting tetanus immunity and providing a platform for preschool-aged vaccination for other antigens. In Malawi, preschool age typically ranges from 3 to 5 years, and by age 6, most children have enrolled in primary school. Administering booster vaccine doses at age 5 can help ensure that children are caught up on vaccinations before they begin their formal education. Periodic multi-antigen catch-up campaigns, like those for measles, could be leveraged to efficiently administer these school-age vaccinations. Studies of TCV co-administration with other vaccines support either a routine or campaign-based strategy.^33^

Alternative vaccination strategies in development include multivalent Salmonella vaccines that incorporate S. *Typhimurium*, *Enteritidis*, and/or *Paratyphi A* antigens, in addition to S. *Typhi,* and would be useful in populations where such pathogens are endemic.

This study's strength lies in its being nested in a randomised trial, maintaining the initial randomisation of vaccine allocations. The inclusion of a control arm enabled meaningful comparisons. Study investigators remained blinded to the original vaccine allocation at enrolment, thereby reducing bias. These factors allowed for the interpretation of our results within the context of the efficacy trial in the same population. Enrolling children from the RCT immunogenicity sub-study facilitated a comprehensive multi-time point immunogenicity analysis, with comparison of results obtained in the same laboratory using the same methodology.

Although the sample size was small and further reduced at day 160–180 due to the TCV campaign, it remains comparable to similar studies, and our findings align with existing knowledge on TCV safety and immunogenicity. It is possible that some children included in the day 160–180 analysis may have received a campaign TCV vaccine dose without the parent's knowledge, such as during community vaccination efforts in schools. However, this is likely to be minimal and non-differential across vaccination arms. Any potential bias would be toward the null, thereby reinforcing the robustness of the observed difference between the boosted and unboosted groups. We were unable to analyse changes in anti-Vi IgA immunogenicity over time due to limited data from the primary immunogenicity study, but the pre- and post-booster vaccination data provide the first results on anti-Vi IgA responses following Vi-TT vaccination in this Malawian population.

Our study demonstrates that a booster dose of Vi-TT administered four years after the primary Vi-TT vaccination is well-tolerated and elicits a robust immune response. The decision to offer booster Vi-TT vaccination—and its timing—should be informed by the local epidemiology of typhoid fever, as well as logistical and cost considerations. These findings are valuable for policymakers at the global and national levels.

## Contributors

NNN, OMN, FM, TM, MAG, MBL, and KMN conceived the study, developed the protocol and standard operating procedures, and managed ethical submissions. NNN, OMN, and VM recruited participants and performed participant follow-up procedures. NNN, OMN, and PDP reported serious adverse events and MBL reviewed them. JO and MFP processed clinical specimens for immunogenicity and generated antibody titers. JMN, BL, NN, OMN, and VM managed study vaccines, performed randomization, and supervised injections. SD developed the statistical analysis plan and conducted analyses. SD and NNN accessed and verified the data. LPJ provided logistical and editorial support. NN, SD, LPJ, and KMN drafted this article. All authors confirm they had full access to the data, accept responsibility for submitting it for publication, and agree to be accountable for all aspects of the work. All authors read and approved the final manuscript. KMN was responsible for the decision to submit the manuscript.

## Data sharing statement

After publication, the authors will provide data that underlie the results reported, after de-identification (text, tables, figures), to researchers who provide a methodologically sound proposal with approved aims. Proposals should be directed to mlaurens@som.umaryland.edu; to gain access, data requestors will need to sign a data use agreement.

## Declaration of interests

NN, SD, LPJ, and MBL receive funding from the TyVAC grant (INV-030857). KMN received funding from the TyVAC grant (INV-030857) through April 2024. MP's laboratory received funding to support this work from INV-030857. KMN is a WHO Strategic Advisory Group of Experts on Immunisation voting member. The authors reported no additional potential competing interests.

## References

[1] Global Burden of Disease Collaborative Network (2022). Global burden of disease study 2021 (GBD 2021) results. https://vizhub.healthdata.org/gbd-results/.

[2] Wang H., Zhang P., Zhao Q., Ma W. (2024). Global burden, trends and inequalities for typhoid and paratyphoid fever among children younger than 15 years over the past 30 years. J Travel Med.

[3] World Health Organization (2019). Typhoid vaccines: WHO position paper, March 2018 - recommendations. Vaccine.

[4] Nampota-Nkomba N., Nyirenda O.M., Khonde L. (2022). Safety and immunogenicity of a typhoid conjugate vaccine among children aged 9 months to 12 years in Malawi: a nested substudy of a double-blind, randomised controlled trial. Lancet Glob Health.

[5] Khanam F., Babu G., Rahman N. (2023). Immune responses in children after vaccination with a typhoid Vi-tetanus toxoid conjugate vaccine in Bangladesh. Vaccine.

[6] Sirima S.B., Ouedraogo A., Barry N. (2021). Safety and immunogenicity of Vi-typhoid conjugate vaccine co-administration with routine 9-month vaccination in Burkina Faso: a randomized controlled phase 2 trial. Int J Infect Dis.

[7] Ouedraogo A., Diarra A., Nébié I. (2023). Durable anti-vi IgG and IgA antibody responses in 15-month-old children vaccinated with typhoid conjugate vaccine in Burkina Faso. J Pediatric Infect Dis Soc.

[8] Sirima S.B., Ouedraogo A., Barry N. (2021). Safety and immunogenicity of co-administration of meningococcal type A and measles-rubella vaccines with typhoid conjugate vaccine in children aged 15-23 months in Burkina Faso. Int J Infect Dis.

[9] Nampota-Nkomba N., Nyirenda O.M., Mapemba V. (2024). Single and two-dose typhoid conjugate vaccine safety and immunogenicity in HIV-exposed uninfected and HIV-unexposed uninfected Malawian children. Hum Vaccin Immunother.

[10] Shakya M., Voysey M., Theiss-Nyland K. (2021). Efficacy of typhoid conjugate vaccine in Nepal: final results of a phase 3, randomised, controlled trial. Lancet Glob Health.

[11] Qadri F., Khanam F., Liu X. (2021). Protection by vaccination of children against typhoid fever with a Vi-tetanus toxoid conjugate vaccine in urban Bangladesh: a cluster-randomised trial. Lancet.

[12] Patel P.D., Patel P., Liang Y. (2021). Safety and efficacy of a typhoid conjugate vaccine in Malawian children. N Engl J Med.

[13] Yousafzai M.T., Karim S., Qureshi S. (2021). Effectiveness of typhoid conjugate vaccine against culture-confirmed Salmonella enterica serotype Typhi in an extensively drug-resistant outbreak setting of Hyderabad, Pakistan: a cohort study. Lancet Glob Health.

[14] Hoffman S.A., LeBoa C., Date K. (2023). Programmatic effectiveness of a pediatric typhoid conjugate vaccine campaign in Navi Mumbai, India. Clin Infect Dis.

[15] Batool R., Tahir Yousafzai M., Qureshi S. (2021). Effectiveness of typhoid conjugate vaccine against culture-confirmed typhoid in a peri-urban setting in Karachi: a case-control study. Vaccine.

[16] Lightowler M.S., Manangazira P., Nackers F. (2022). Effectiveness of typhoid conjugate vaccine in Zimbabwe used in response to an outbreak among children and young adults: a matched case control study. Vaccine.

[17] Qadri F., Khanam F., Zhang Y. (2024). Five-year vaccine protection following a single dose of Vi-tetanus toxoid conjugate vaccine in Bangladeshi children: a cluster randomised trial. SSRN.

[18] Patel P.D., Liang Y., Meiring J.E. (2024). Efficacy of typhoid conjugate vaccine: final analysis of a 4-year, phase 3, randomised controlled trial in Malawian children. Lancet.

[19] Qamar F.N., Qureshi S., Haq Z. (2024). Longevity of immune response following a single dose of typhoid conjugate vaccine against Salmonella Typhi among children in Hyderabad, Pakistan. Int J Infect Dis.

[20] Meiring J.E., Laurens M.B., Patel P. (2019). Typhoid vaccine acceleration consortium Malawi: a Phase III, randomized, double-blind, controlled trial of the clinical efficacy of typhoid conjugate vaccine among children in Blantyre, Malawi. Clin Infect Dis.

[21] Kulkarni P.S., Potey A.V., Bharati S. (2024). The safety and immunogenicity of a bivalent conjugate vaccine against Salmonella enterica Typhi and Paratyphi A in healthy Indian adults: a phase 1, randomised, active-controlled, double-blind trial. Lancet.

[22] Tapia M.D., Pasetti M.F., Cuberos L. (2006). Measurement of tetanus antitoxin in oral fluid: a tool to conduct serosurveys. Pediatr Infect Dis J.

[23] Medise B.E., Soedjatmiko S., Rengganis I. (2019). Six-month follow up of a randomized clinical trial-phase I study in Indonesian adults and children: safety and immunogenicity of Salmonella typhi polysaccharide-diphtheria toxoid (Vi-DT) conjugate vaccine. PLoS One.

[24] Bijukchhe S.M., Gurung M., Pokhrel B. (2024). Immune responses to typhoid conjugate vaccine in a two dose schedule among Nepalese children <2 years of age. Vaccine.

[25] Capeding M.R., Tadesse B.T., Sil A. (2022). Immune persistence and response to booster dose of Vi-DT vaccine at 27.5 months post-first dose. NPJ Vaccines.

[26] Vadrevu K.M., Raju D., Rani S. (2021). Persisting antibody responses to Vi polysaccharide-tetanus toxoid conjugate (Typbar TCV®) vaccine up to 7 years following primary vaccination of children < 2 years of age with, or without, a booster vaccination. Vaccine.

[27] Mohan V.K., Varanasi V., Singh A. (2015). Safety and immunogenicity of a Vi polysaccharide-tetanus toxoid conjugate vaccine (Typbar-TCV) in healthy infants, children, and adults in typhoid endemic areas: a multicenter, 2-cohort, open-label, double-blind, randomized controlled phase 3 study. Clin Infect Dis.

[28] Meiring J.E., Shakya M., Khanam F. (2021). Burden of enteric fever at three urban sites in Africa and Asia: a multicentre population-based study. Lancet Glob Health.

[29] World Health Organization (2018). Tetanus vaccines: WHO position paper, February 2017 - recommendations. Vaccine.

[30] World Health Organization (2021). Table 1: summary of WHO position papers - recommendations for routine immunization. https://www.who.int/publications/m/item/table-1-who-recommendations-for-routine-immunization.

[31] Al-Qaisi T.S., Abumsimir B. (2023). Vaccination strategies, the power of the unmatched double hits. Future Sci OA.

[32] Burrows H., Antillón M., Gauld J.S. (2023). Comparison of model predictions of typhoid conjugate vaccine public health impact and cost-effectiveness. Vaccine.

[33] Nampota-Nkomba N., Carey M.E., Jamka L.P., Fecteau N., Neuzil K.M. (2023). Using typhoid conjugate vaccines to prevent disease, promote health equity, and counter drug-resistant typhoid fever. Open Forum Infect Dis.

