# Supplementary material for: Immunogenicity and reactogenicity of a booster dose of a typhoid conjugate vaccine (TCV) in Malawian pre-school children
Source: eClinicalMedicine. 2025 Feb 12;81:103100. doi: 10.1016/j.eclinm.2025.103100 (PMC11872497; doi:10.1016/j.eclinm.2025.103100)
Supplement: Supplementary Figures and Tables [file mmc1.docx]

| Supplemental Table 1: Anti-Vi IgG before, 28 and 160-180 days after Vi-TT vaccination given 4+ years after receipt of first Vi-TT or Men-A in the intention to treat population. | | | | |
| --- | --- | --- | --- | --- |
|  | Booster-TCV | | 1st-TCV | |
|  | *n or n/N* | *GMT or % (95% CI)* | *n or n/N* | *GMT or % (95% CI)* |
| Geometric Mean Titer (GMT,EU/mL) |  | *GMT (95%CI)* |  | *GMT (95%CI)* |
| Day 0 | 71 | 18·8 (15·2-23·2) | 64 | 5·7 (4·6-7·2) |
| Day 28 | 71 | 6794·2 (5738·2-8044·6) | 63 | 2854·8 (2381·7-3421·9) |
| Day 160-180* | 45 | 486·7 (376·0-630·0) | 40 | 299·5 (214·5-418·2) |
| Median (Interquartile range) |  | *Median (q1, q3)* |  | *Median (q1, q3)* |
| Day 0 | 71 | 18·1 (12·7, 30·8) | 64 | 3·7 (3·7, 7·5) |
| Day 28 | 71 | 6739·5 (4288·3, 10822·1) | 63 | 2834·5 (1372·5, 4912·7) |
| Day 160-180* | 45 | 439·1 (272·3, 885·8) | 40 | 257·4 (154·1, 447·8) |
| Geometric Mean Fold Rise (GMFR) |  | *GMFR (95%CI)* |  | *GMFR (95%CI)* |
| Day 0 to 28 | 70 | 370·1 (289·4-473·3) | 63 | 493·9 (369·0-661·0) |
| Day 0 to 160-180* | 44 | 25·0 (18·9-32·9) | 40 | 52·7 (36·6-75·9) |
| Seroconversion ≥ 4-fold increase from |  | *% (95% CI)* |  | *% (95% CI)* |
| Day 0 to 28 | 70/70 | 100·0 (94·9-100·0) | 62/63 | 98·4 (91·5-100·0) |
| Day 0 to 160-180* | 42/44 | 95·5 (84·5-99·4) | 40/40 | 100·0 (91·2-100·0) |
| n=number of participants. CI=confidence interval. Vi-TT: Vi polysaccharide tetanus toxoid conjugate vaccine. Men-A: meningococcal polysaccharide tetanus toxoid conjugate vaccine. EU: ELISA units. *Participants who received TCV as part of the Malawi national campaign were excluded. | | | | |

| Supplemental Table 2: Anti-Vi IgA before, 28 and 160-180 days after Vi-TT vaccination given 4+ years after receipt of first Vi-TT or Men-A in the intention to treat population. | | | | |
| --- | --- | --- | --- | --- |
|  | Booster-TCV | | 1st-TCV | |
|  | *n or n/N* |  | *n or n/N* |  |
| Geometric Mean Titer (GMT,EU/mL) |  | *GMT (95%CI)* |  | *GMT (95%CI)* |
| Day 0 | 71 | 2·6 (2·2-3·1) | 64 | 1·7 (1·5-1·9) |
| Day 28 | 71 | 126·0 (103·1-153·8) | 63 | 87·6 (69·7-110·2) |
| Day 160-180* | 45 | 60·8 (44·7-82·7) | 40 | 45·5 (33·4-62·1) |
| Median (Interquartile range ) |  | *Median (q1, q3)* |  | *Median (q1, q3)* |
| Day 0 | 71 | 1·6 (1·6, 4·.3) | 64 | 1·6 (1·6, 1·6) |
| Day 28 | 71 | 124·7 (78·8, 799·5) | 63 | 102·6 (56·6, 152·9) |
| Day 160-180* | 45 | 58·5 (37·0, 107·7) | 40 | 49·2 (23·6, 71·3) |
| Geometric Mean Fold Rise (GMFR) |  | *GMFR (95%CI)* |  | *GMFR (95%CI)* |
| Day 0 to 28 | 70 | 48·7 (38·4-61·6) | 63 | 52·4 (39·6-69·3) |
| Day 0 to 160-180* | 44 | 24·1 (17·9-32·4) | 40 | 28·6 (20·9-39·1) |
| Seroconversion ≥ 4-fold increase from |  | *% (95% CI)* |  | *% (95% CI)* |
| Day 0 to 28 | 69/70 | 98·6 (92·3-100·0) | 61/63 | 96·8 (89·0-99·6) |
| Day 0 to 160-180* | 43/44 | 97·7 (88·0-100·0) | 39/40 | 97·5 (86·8-100·0) |
| n=number of participants. CI=confidence interval. Vi-TT: Vi polysaccharide tetanus toxoid conjugate vaccine. Men-A: meningococcal polysaccharide tetanus toxoid conjugate vaccine. EU: ELISA units. *Participants who received TCV as part of the Malawi national campaign were excluded. | | | | |

**wSupplemental Figure 1:** Anti-Vi IgG GMT before, 28 and 160-180 days after Vi-TT vaccination given 4+ years after receipt of first Vi-TT or Men-A in the per-protocol population


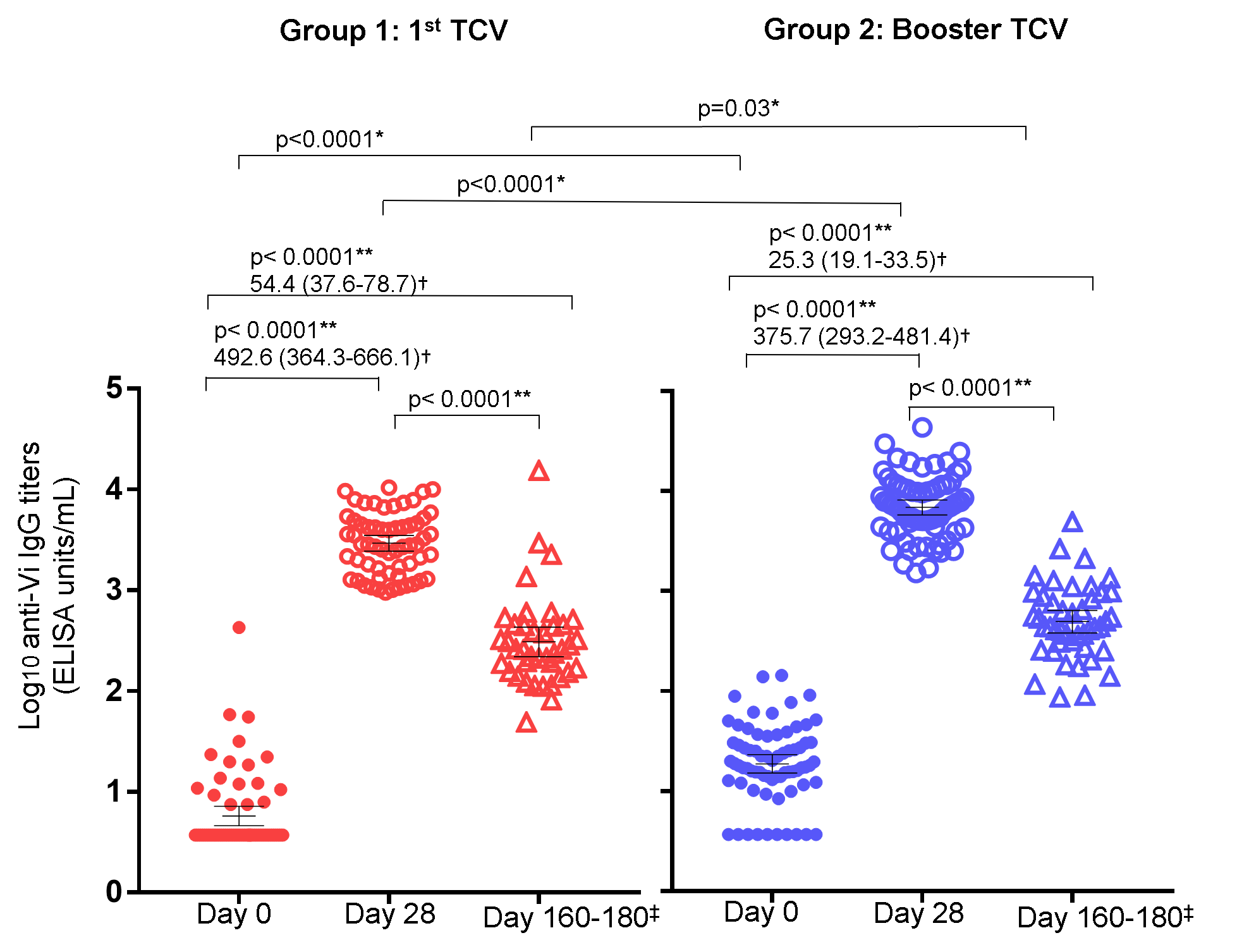


^‡^Participants who received a TCV dose during the 2023 national campaign have been excluded. . ^†^Geometric Mean Fold Rise (95%CI). *Using two sample t-test with unequal variances on log10 transformed data. **Using paired t-test on log10 transformed data. Vi-TT: Vi polysaccharide tetanus toxoid conjugate vaccine. Men-A: meningococcal polysaccharide tetanus toxoid conjugate vaccine. CI: confidence interval.

**Supplemental Figure 2:** Anti-Vi IgA GMT before, 28 and 160-180 days after Vi-TT vaccination given 4+ years after receipt of first Vi-TT or Men-A in the per-protocol population


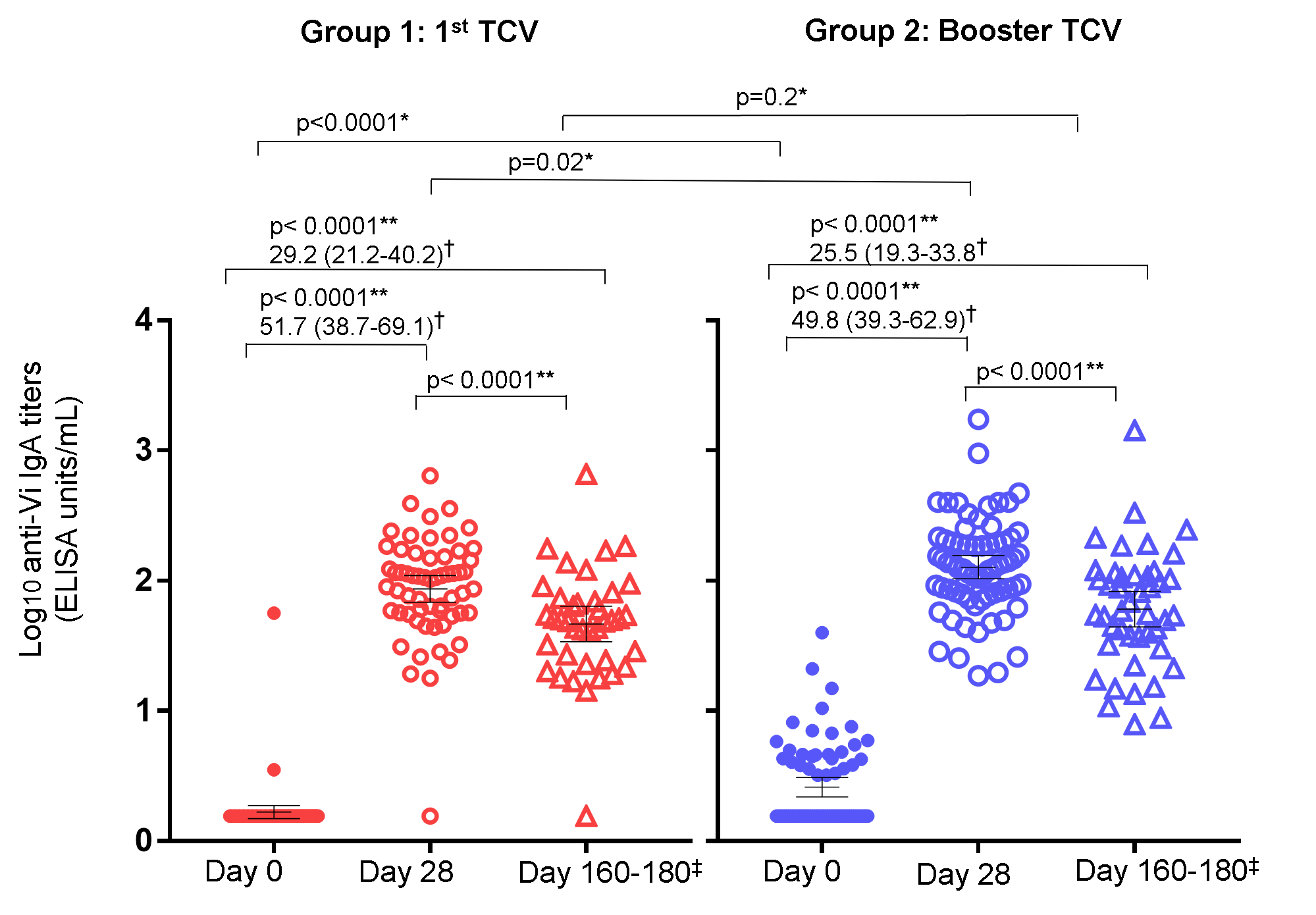


^‡^Participants who received a TCV dose during the 2023 national campaign have been excluded. . ^†^Geometric Mean Fold Rise (95%CI). *Using two sample t-test with unequal variances on log10 transformed data. **Using paired t-test on log10 transformed data. Vi-TT: Vi polysaccharide tetanus toxoid conjugate vaccine. Men-A: meningococcal polysaccharide tetanus toxoid conjugate vaccine. CI: confidence interval.

**Supplemental Figure 3:** Anti-Tetanus IgG GMT before, 28 and 160-180 days after Vi-TT vaccination given 4+ years after receipt of first Vi-TT or Men-A in the per-protocol population


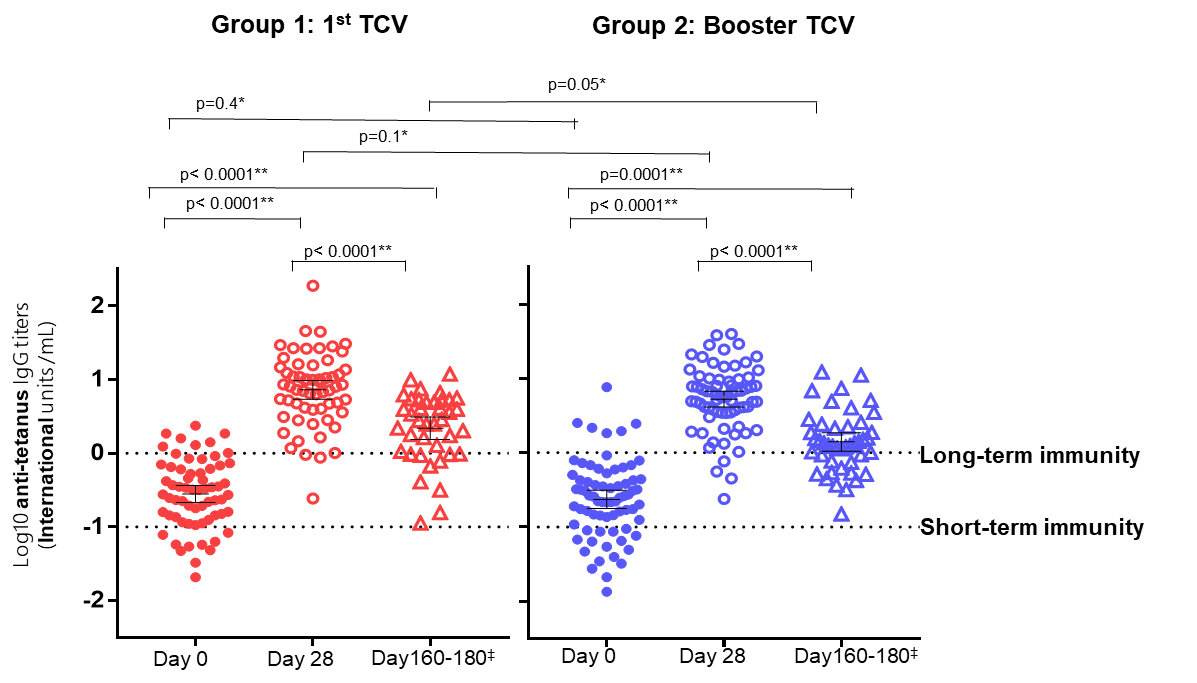


^‡^Participants who received a TCV dose during the 2023 national campaign have been excluded. *Using two sample t-test with unequal variances on log10 transformed data. **Using paired t-test on log10 transformed data. Short term immunity: ≥ 0·1 IU/mL. Long term immunity: ≥ 1 IU/m
